# Association between gastrointestinal events and osteoporosis treatment initiation in women diagnosed with osteoporosis in France: a retrospective analysis

**DOI:** 10.1186/s12891-016-1041-8

**Published:** 2016-04-30

**Authors:** Bernard Cortet, Ankita Modi, Jackson Tang, Chun-Po Steve Fan, Shiva Sajjan, Jessica Papadopoulos Weaver

**Affiliations:** Rheumatology Department, University Hospital of Lille, Lille Cedex, France; Merck & Co, Inc., Kenilworth, NJ 07033 USA; Asclepius Analytics Ltd, Brooklyn, NY USA; Hospital for Sick Children, Toronto, Canada; Center for Observational and Real-World Evidence, Mailstop: CRB-205 Merck & Co., Inc., 600 Corporate Drive, Lebanon, NJ 08833 USA

**Keywords:** Bisphosphonates, Gastrointestinal, Osteoporosis, Postmenopausal, Prescribing

## Abstract

**Background:**

A substantial portion of women diagnosed with osteoporosis (OP) do not initiate pharmacotherapy to reduce fracture risk. In clinical practice, gastrointestinal (GI) events have been linked with OP therapy discontinuation. However, there is limited research examining GI events as barrier to treatment initiation following an OP diagnosis. The objective of this study was to examine the association between gastrointestinal (GI) events and osteoporosis (OP) treatment initiation among post-menopausal women diagnosed with osteoporosis in France.

**Methods:**

A retrospective claims analysis of the Mediplus France database during 1997 to 2010 identified women aged ≥ 55 with an OP diagnosis and without prior OP treatment (first diagnosis date was defined as the index date). GI events were identified during the 1 year pre-index and up to 1 year post-index. OP treatment initiation post-index was identified based on the presence of claims for any bisphosphonate (BIS) or non-BIS OP medication within 1 year post-index. Multivariate models (logistic regression, Cox proportional hazards regression and discrete choice) adjusted for pre-index patient characteristics were used to assess the association of pre- and post-index GI events with the likelihood of initiating OP treatment, and the type of treatment initiated (BIS vs. non-BIS).

**Results:**

A total of 10,292 women (mean age 70.3 years) were identified; only 25 % initiated OP treatment. Post-index GI events occurred in 11.5 % of patients, and were associated with a 75.7 % lower likelihood of initiating OP treatment. Among treated patients, a discrete choice model estimated that patients with post-index GI events were 34.6 % less likely to receive BIS vs non-BIS as compared to patients without post-index GI events.

**Conclusion:**

Among women aged ≥ 55 years with an OP diagnosis, post-index GI events were associated with a lower likelihood of OP treatment initiation.

**Electronic supplementary material:**

The online version of this article (doi:10.1186/s12891-016-1041-8) contains supplementary material, which is available to authorized users.

## Background

There were an estimated 22 million women and 5.5 million men aged 50–84 with osteoporosis (OP) in the European Union (EU) in 2010 [1], and the numbers are projected to rise 23 % by 2025. There were also 3.5 million fractures in this population, two-thirds of which occurred in women [1]. In France, the INSTANT study reported OP prevalence among women aged 45 and older to be around 10 %, or 1.1 million women in 2006 [2], although other estimates put the 2010 prevalence at 3 million and project it to rise to 3.4 million by 2020 [3]. The associated cost and health burden of OP is substantial. The overall cost associated with OP in the EU in 2010 was estimated to be €37 billion, 66 % of which was attributed to treating incident fractures [1]. In addition, OP-related fractures negatively impact health-related quality of life [4–6], and increase the risk of mortality [7, 8].

Treatment for OP can reduce the risk of fracture, and French guidelines for management of OP recommend pharmacotherapy for those at risk [9]. Several therapies were available for use from 1997 to 2010. Bisphosphonates (BIS: alendronate, ibandronate, risedronate, zoledronic acid) are the most widely used therapy in the field of OP treatment. Non-BIS, such as denosumab, raloxifene, teriparatide, and strontium ranelate, are also used to treat OP.

Estimates of OP treatment penetration in France vary widely. The INSTANT study reported that 61 % of OP patients were treated, and levels of treatment penetration increased with age [2]. In an observational study of general practice-recruited women diagnosed with OP, 97 % were receiving treatment [10], although the study was limited to patients who had been followed for at least 2 years. There have been studies of OP treatment rates among patients diagnosed with OP in other areas of Europe, including a study in Germany that found that 22 % were treated [11], a study in Austria that reported only 7 % of nursing home residents received treatment [12], and a study in Switzerland indicating that 24 % of women were “adequately” treated with a bone active substance [13]. While these studies vary in setting and study type, they consistently report significant under-treatment of OP.

The barriers to OP treatment are not fully understood, but there are several potential reasons for low treatment rates. Patients may not take prescribed medication because they may not fully understand OP [14], or they may be skeptical of the effectiveness of medication [15] or have concerns over side-effects [16]. Additionally, patients may underestimate their risk for fracture [17, 18] and assume that treatment is unnecessary. Physicians who fail to prescribe OP therapy may not consider OP a priority compared with other diseases present in their patients, or may assume that ongoing treatment of OP will be handled by another physician [19–21]. Gastrointestinal (GI) intolerance has also been cited as a reason by physicians for not prescribing BIS [22], and as a reason for discontinuing treatment among patients receiving treatment [23–25], although the risk of a GI event is often a function of pre-existing GI conditions [26]. There is a need to further explore how GI events may impact treatment initiation among osteoporotic patients. The objective of this study was to examine OP treatment initiation patterns and the association between GI events and OP treatment initiation among post-menopausal women with OP in France.

## Methods

### Study design and data source

This study was a retrospective claims analysis of patients in the Mediplus France database (now known as the IMS Disease Analyzer database: France) during 1997 to 2010. This physician-based database contains approximately 1.1 million patients and includes demographic, medical, and pharmaceutical claims, as well as the results of lab tests. The identification of OP and other diseases is available from International Classification of Diseases, Tenth Revision (ICD-10) diagnosis codes. Prescription information uses the ATC-4 coding convention. All data were de-identified to preserve patient and physician anonymity.

### Patient identification

Women with at least one OP diagnosis (ICD-10 code: M80, M81) during 1997 to 2010 were identified. The date of the first claim with an OP diagnosis during this interval was defined as the index date. The pre-index period (baseline) was the 12-month period prior to the index date; the post-index period was the 12-month period following the index date. To be included, patients were required to be 55 years or older as of the index date, naïve to OP treatment pre-index, and have continuous enrollment for 1 year before and after the index date. Patients with evidence of Paget’s disease (ICD-10 code: M88) at any time in their claims history, a malignant neoplasm (ICD-10 code: C00-C42, C44-C96, D00-D09, D37-D49) at any point (pre- or post-index), or with estrogen use during the pre-index period were excluded.

### Measures

Patient characteristics captured during the pre-index period included age, OP-related comorbidities, medication use (glucocorticoids, non-steroidal anti-inflammatory drugs [NSAIDs], gastro-protective agents), fractures and the Charlson Comorbidity Index (CCI) score [27]. The CCI is an aggregate score based on 17 medical conditions, and serves as a measure of overall disease burden. The occurrence and severity of GI events were also assessed during the pre-index period and included a broad range of upper and lower GI conditions identified via 79 distinct ICD-10 codes (e.g., abdominal pain, acute gastritis, dysphagia, esophagitis, esophageal, gastric and duodenal ulcers, gastro-esophageal reflux disease, heartburn, nausea, vomiting). The codes used to identify GI events and fractures are shown in the Additional file 1.

Treatment during the post-index period was defined as evidence of any OP treatment (oral, injectable, infused or intranasal) within one year after the index date. For patients identified as receiving treatment, the date of treatment initiation was defined as the date of the first indicated therapy, and the initial treatment type was classified as either BIS or non-BIS. BIS medications included alendronate, ibandronate, risedronate, and zoledronic acid, while non-BIS medications were raloxifene, calcitonin, teriparatide, strontium ranelate, and parathyroid hormone (although calcitonin is not officially authorized in France, it is still prescribed). The same GI events identified during the pre-index period were also assessed during the post-index period (Additional file 1). The observation period for post-index GI events was the entire 12-month post-index period for patients who did not initiate treatment. For patients who did initiate treatment, post-index GI events were identified from the index date up until the date of treatment initiation.

### Statistical analysis

Summary measures were calculated for all baseline characteristics, and the count and percentage of patients with pre- and post-index GI events, post-index OP treatment initiation, and the type of OP treatment, were calculated. Comparisons of summary measures were made between cohorts based on pre- and post-index GI events as well as the type of post-index OP treatment initiated (BIS vs non-BIS). Differences were compared using Wilcoxon rank-sum tests for continuous measures and chi-square tests for categorical measures.

Multivariate analyses were employed to examine the association between post-index GI events and both treatment initiation and type of OP treatment initiated (BIS vs non-BIS) during the post-index period. Logistic regression was used to assess the association between post-index GI events and likelihood of starting any OP treatment adjusted for pre-index GI events (including an interaction term with post-index GI events), age, pre-index CCI score, and pre-index medication use (gastro-protective agents, NSAIDs, glucocorticoids). The association between post-index GI events and likelihood of initiating with BIS vs non-BIS (among patients who initiated treatment) was examined in a separate logistic regression model with the same adjustment variables. Since logistic regression does not account for the varying length of the exposure period, and given that patients could have different lengths of follow-up, time-varying Cox proportional hazards regression was also utilized to allow for consideration of the timing of post-index GI events on the likelihood of treatment initiation. The Cox proportional hazards model was stratified by the presence or absence of pre-index GI events and was adjusted for age, pre-index CCI score and pre-index medication use. Finally, among treated patients, a discrete choice model with conditional logit was employed to model the association between post-index GI events and receipt of BIS versus non-BIS among patients who initiated treatment, adjusted for age, pre-index GI events, pre-index CCI score, and pre-index medication use.

## Results

### Patient sample and baseline characteristics

**Fig. 1:**
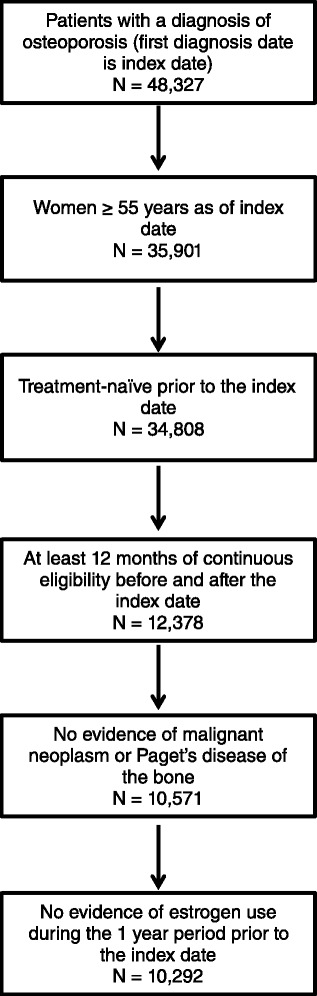
Study sample selection

A total of 10,292 patients were identified for analysis (Fig. 1). The mean (SD) age was 70.3 (8.2) years and the mean (SD) CCI score was 0.92 (1.22) (Table 1). The most common comorbid conditions were hypertension (78.8 % of patients), depression (23.9 %), and diabetes (16.0 %). Pre-index GI events occurred in 1452 (14.1 %) of patients and 7.6 % experienced a pre-index fracture. The percentage of patients with at least one claim for NSAIDs was 74.2 %, while 42.6 and 38.7 % had at least one claim for glucocorticoids and gastro-protective agents, respectively. There were several significant differences in characteristics between patients with and patients without post-index events, including a higher rate of pre-index GI events (35.1 % vs. 11.4 % *P* < 0.001) and gastro-protective agent use (46.8 % vs. 37.7 %, *P* < 0.001) among patients with post-index events. Patients with post-index event were also more like to have comorbid hypertension, depression and diabetes, and to use NSAIDs and glucocorticoids. Among patients who initiated OP treatment (*n* = 2603), baseline characteristics were similar among patients initiating with BIS compared with patients initiating with non-BIS with the exception that BIS initiators had a higher CCI score and greater rate of hypertension and gastro-protective agent use than non-BIS initiators.

**Table 1:** Patient baseline characteristics

		Post-index GI status *n* = 10,292			Treatment initiated (treated patients only) *n* = 2603		
Baseline characteristic	All patients (*n* = 10,292)	With post-index GI events (*n* = 1180)	Without post-index GI events (*n* = 9112)	*p* value	Initiated BIS treatment (*n* = 2422)	Initiated non-BIS treatment (*n* = 181)	*p* value
Age, mean (SD)	70.3 (8.2)	71.4 (7.5)	70.2 (8.3)	0.010	71.5 (9.4)	70.7 (9.2)	0.074
Charlson comorbidity index score, mean (SD)	0.92 (1.22)	1.12 (1.04)	0.89 (0.76)	<.001	1.26 (1.35)	0.71 (1.04)	<.001
Common OP-related comorbidities, *n* (%)							
Chronic inflammatory bowel disease	59 (0.6)	7 (0.6)	52 (0.6)	0.995	17 (0.7)	1 (0.6)	0.972
Chronic inflammatory joint disease	623 (6.1)	95 (8.1)	528 (5.8)	<.001	109 (4.5)	3 (1.7)	0.211
Celiac disease	2 (0.0)	0 (0.0)	2 (0.0)	0.856	0 (0.0)	0 (0.0)	NA
Diabetes (type 1 or 2)	1649 (16.0)	246 (20.8)	1403 (15.4)	0.003	191 (4.5)	14 (7.7)	0.760
Depression	2456 (23.9)	497 (42.1)	1959 (21.5)	<.001	254 (10.5)	19 (10.5)	0.924
Chronic kidney disease	120 (1.2)	20 (1.7))	100 (1.1)	0.045	3 (0.1)	1 (0.6)	0.215
Fatigue	434 (4.2)	88 (7.5)	346 (3.8)	<.001	36 (1.5)	2 (1.1)	0.398
Hypertension	8113 (78.8)	1042 (88.3)	7071 (77.6)	<.001	1223 (50.5)	73 (40.3)	0.002
GI mucositis & urination problems	543 (5.3)	106 (9.0)	437 (4.8)	<.001	95 (3.9)	6 (3.3)	0.075
Hyperparathyroidism	36 (0.3)	4 (0.3)	32 (0.4)	0.879	11 (0.5)	1 (0.6)	0.510
Vitamin D deficiency	3 (0.0)	0 (0.0)	3 (0.0)	0.785	0 (0.0)	0 (0.0)	NA
OP-related fractures, *n* (%)	778 (7.6)	138 (11.7)	640 (7.0)	0.344	304 (12.6)	38 (21.0)	0.231
GI-related drug use, *n* (%)							
Glucocorticoids	4382 (42.6)	728 (61.7)	3654 (40.1)	<.001	509 (21.0)	32 (17.7)	0.180
NSAIDs	7633 (74.2)	1036 (87.8)	6597 (72.4)	<.001	1017 (42.0)	72 (39.8)	0.155
Gastro-protective agents	3987 (38.7)	552 (46.8)	3435 (37.7)	<.001	654 (27.0)	36 (19.9)	0.004
GI events (pre-index), *n* (%)	1452 (14.1)	414 (35.1)	1038 (11.4)	<.001	304 (12.6)	38 (21.0)	0.231

*GI* gastrointestinal, *NA* not applicable, *NSAIDs* non-steroidal anti-inflammatory drugs

Treatment initiation status and the type of OP treatment initiated are shown in Table 2. Only 2603 patients (25.3 %) initiated OP treatment during the post-index period. The large majority of these patients (93.0 %) initiated using BIS. The most frequently used BIS was alendronate (1235 of 2422 patients, 51.0 %), while the most common non-BIS was raloxifene (97 of 181, 53.6 %).

**Table 2:** Treatment patterns within one year of osteoporosis diagnosis

	All patients (*n* = 10,292)	
	*n*	%
No treatment	7689	74.7
Bisphosphonates	2422	23.5
Alendronate	1265	12.3
Zoledronic acid	720	7.0
Risedronate	312	3.0
Ibandronate	125	1.2
Non-bisphosphonates	181	1.8
Raloxifene	97	0.9
Strontium ranelate	43	0.4
Calcitonin	41	0.4
Teriparatide	0	0.0
Parathyroid hormone	0	0.0
Mean time until treatment (days)	Mean	SD
Bisphosphonate initiation (*n* = 2422)	48.9	81.2
Non-bisphosphonate initiation (*n* = 181)	40.8	78.4

*SD* standard deviation

The distribution of patients by the presence/absence of GI events and their treatment initiation status is shown in Table 3. Post-index GI events occurred in 1180 (11.5 %) patients, of which 35.1 % (414 of 1180) had pre-index GI events. Only 7.4 % (766 of 10,292) of patients without pre-index GI events had post-index events. Among those not receiving treatment post-index (7689), a total of 1110 (14.4 %) had pre-index GI events (1110 patients representing 734 patients with no post-index GI event and 376 patients with a post-index GI event). A total of 304 patients of 2422 (12.6 %) initiating BIS treatment and 38 of 181 (21.0 %) initiating non-BIS had pre-index GI events. Post-index GI events occurred in 13.0 % (998 of 7689) of those who did not initiate treatment, in 6.4 % (154 of 2422) of those who initiated treatment with a BIS, and in 15.5 % (28 of 181) of those who initiated with a non-BIS. Overall, treatment penetration was 23.6 % for those with pre-index GI events and 25.6 % for those without pre-index GI events. Among treated patients, BIS was initiated in 88.9 % of patients with pre-index GI events, compared with 93.7 % of patients without pre-index GI events.

**Table 3:** Distribution of patients by pre- and post-index GI events

Presence of GI events		Treatment within 1 year post-index			
Pre-index	Post-index	No treatment *n* (%)	BIS *n* (%)	Non-BIS *n* (%)	Total *n* (%)
No	No	5957 (73.8)	1992 (24.7)	125 (1.5)	8074 (78.4)
Yes	No	734 (70.7)	276 (26.6)	28 (2.7)	1038 (10.1)
No	Yes	622 (81.2)	126 (16.4)	18 (2.3)	766 (7.4)
Yes	Yes	376 (90.8)	28 (6.8)	10 (2.4)	414 (4.0)
Total		7689 (74.7)	2422 (23.5)	181 (1.8)	10,292

*BIS* bisphosphonate, *GI* gastrointestinal

Among patients with post-index GI events, 15.4 % (182 of 1180) initiated OP treatment (84.6 % of those initiated with BIS) (Table 3). By comparison, 26.6 % (2421 out of 9112) of those without a post-index GI event initiated treatment (93.6 % with BIS). Only 9.2 % of patients with GI events during both the pre- and post-index period initiated OP treatment.

Results from the logistic regression analysis examining the association between post-index GI events and treatment initiation are shown in Table 4. This analysis indicates that among patients with no pre-index GI events, the occurrence of post-index GI events was associated with 70.7 % lower odds of initiating OP treatment (odds ratio [OR]: 0.293]; 95 % confidence interval [CI]: 0.25–0.34). Among patients with a pre-index GI event, a post-index GI event was associated with 74.3 % reduced odds of treatment initiation (OR: 0.257; 95 % CI: 0.20–0.31). In addition to post-index GI events, certain patient characteristics were significantly associated with likelihood of treatment initiation. The likelihood of treatment initiation increased by 31.9 % for patients aged 65–74 (vs. 55–64) and by 53.8 % for patients aged 75–84 (vs. 55–64). Similarly, pre-index medication use increased odds of initiating treatment by 75.5 % for gastro-protective agents, 33.7 % for NSAIDs and 25.4 % for glucocorticoids. Higher pre-index CCI score was associated with 7.1 % lower odds of treatment initiation. In the logistic regression analysis limited to patients who initiated treatment (Table 5), post-index GI events lowered the odds of receiving BIS (vs. non-BIS) by 40.9 % (OR: 0.591; 95 % CI: 0.55–0.64) among patients with no pre-index events. A lesser likelihood of receiving BIS was also apparent for patients with pre-index GI events (OR: 0.631; 95 % CI: 0.58–0.68). Patients characteristics associated with significantly greater odds of initiating with BIS were age group 75–84 vs 55–64 (57.6 % greater odds) and pre-index use of gastro-protective agents (69.3 % greater odds).

**Table 4:** Logistic regression analysis of association between GI events and treatment initiation

Effect	Initiated any treatment vs. no treatment initiated		
Post-index GI event (ref: no post-index GI event)	Odds ratio	95 % CI	*p* value
Among patients without pre-index GI problems	0.293	(0.25, 0.34)	<0.0001
Among patients with pre-index GI problems	0.257	(0.20, 0.31)	<0.0001
Age group			
65–74 vs. 55–64	1.319	(1.14, 1.47)	0.0024
75–84 vs. 55–64	1.538	(1.26, 1.72)	<0.0001
85+ vs. 55–64	1.271	(1.05, 1.63)	0.1432
Pre-index medication use			
Gastro-protective agents	1.755	(1.58, 1.95)	<0.0001
NSAIDs	1.337	(1.23, 1.45)	<0.0001
Glucocorticoids	1.254	(1.13, 1.39)	<0.0001
Charlson comorbidity index score	0.929	(0.89, 0.97)	0.0093

*GI* gastrointestinal, *NSAIDs* non-steroidal anti-inflammatory drugs

*CI* confidence interval

**Table 5:** Logistic regression analysis of association between GI events and type of osteoporosis treatment initiated

Effect	Initiated bisphosphonates vs. initiated non-bisphosphonates		
Post-index GI event (ref: no post-index GI event)	Odds ratio	95 % CI	*p* value
Among patients without pre-index GI problems	0.591	(0.55, 0.64)	<0.0001
Among patients with pre-index GI problems	0.631	(0.58, 0.68)	<0.0001
Age group			
65–74 vs. 55–64	1.393	(1.00, 1.95)	0.0603
75–84 vs. 55–64	1.576	(1.18, 2.01)	<0.0001
85+ vs. 55–64	1.154	(0.77, 1.75)	0.2332
Pre-index medication use			
Gastro-protective agents	1.693	(1.21, 2.38)	<0.0001
NSAIDs	1.094	(0.86, 1.39)	0.3443
Glucocorticoids	1.066	(0.78, 1.46)	0.4532
Charlson comorbidity index score	1.004	(0.88, 1.15)	0.3594

*CI* confidence interval

The time-varying Cox regression model results paralleled the logistic regression results (Table 6). Patients with post-index GI events were 75.7 % less likely to initiate treatment (hazard ratio: 0.243; 95 % CI: 0.22–0.27). Similar to the logistic regression model results, characteristics associated with greater likelihood of initiating treatment were older age group (23.4–44.4 % greater odds) and pre-index use of gastro-protective agents (62.1 % greater odds), NSAIDs (28.7 % greater odds) and glucocorticoids (22.3 % greater odds). Higher pre-index CCI score was associated 8.7 % lower odds of initiating treatment.

**Table 6:** Time-varying Cox regression analysis of association between GI events and treatment initiation

Effect	Estimated hazard ratio	95 % CI	*p* value
Post-index GI event (ref: no post-index GI event)	0.243	(0.22, 0.27)	<0.0001
Age group			
65–74 vs. 55–64	1.274	(1.16, 1.40)	<0.0001
75–84 vs. 55–64	1.444	(1.31, 1.60)	<0.0001
85+ vs. 55–64	1.234	(1.06, 1.43)	0.0023
Pre-index medication use			
Gastro-protective agents	1.621	(1.52, 1.79)	<0.0001
NSAIDs	1.287	(1.20, 1.38)	<0.0001
Glucocorticoids	1.223	(1.14, 1.68)	<0.0001
Charlson comorbidity index score	0.913	(0.85, 0.98)	0.0034

*GI* gastrointestinal, *NSAIDs* non-steroidal anti-inflammatory drugs

*CI* confidence interval

The results of the discrete choice model among patients who initiated treatment are shown in Table 7. Consistent with the logistic regression model results, post-index GI events were associated with 34.6 % lower odds of initiating treatment with BIS compared with non-BIS (OR: 0.654; 95 % CI: 0.57–0.74) and pre-index GI events reduced the likelihood of receiving BIS by 15.4 % (0.846; 95 % CI: 0.74 – 0.95). Older age and pre-index use of gastro-protective agents were associated with a lower likelihood of initiating with BIS vs. non-BIS. Specifically, when compared with those aged 55–64 years, patients aged 75–84 years were 21.5 % less likely to initiate with BIS. Patients who had used gastro-protective agents during the pre-index period were 31.6 % less likely to initiate with BIS vs. non-BIS.

**Table 7:** Discrete choice model of association between GI events and receipt of bisphosphonate treatment (versus non-bisphosphonate)^a^

Effect	Estimated odds ratio	95 % CI	*p* value
Pre-index GI event (ref: no pre-index GI event)	0.846	(0.74, 0.95)	0.0009
Post-index GI event (ref: no post-index GI event)	0.654	(0.57, 0.74)	<0.0001
Age group			
65–74 vs. 55–64	0.962	(0.87, 1.06)	0.0613
75–84 vs. 55–64	0.785	(0.71, 0.87)	0.0023
85+ vs. 55–64	0.720	(0.54, 0.97)	0.0624
Pre-index Medication Use			
Gastro-protective agents	0.684	(0.62, 0.76)	<0.0001
NSAIDs	1.066	(0.97, 1.17)	0.1835
Glucocorticoids	0.982	(0.88, 1.09)	0.7334
Charlson comorbidity index score	0.961	(0.92, 1.01)	0.0963

*NSAIDs* non-steroidal anti-inflammatory drugs

^a^Among patients who initiated treatment

*CI* confidence interval

## Discussion

In this study, we examined the association between the presence of GI events and the initiation of OP treatment. We found that only 25 % of patients initiated OP treatment in the first year after their OP diagnosis. Patients experiencing post-index GI events were 71 to 74 % less likely to initiate OP treatment. Among patients who initiated treatment, post-index GI events reduced the likelihood of treatment with BIS by 35 %.

The level of treatment penetration among the patients in this study is lower than in some previous studies. The French INSTANT study reported treatment penetration of 61 % [2]; however, INSTANT required bone densitometry which may have restricted eligibility to patients who were more actively managed. Our study period (1997–2010) spans the period before and after bone densitometry scans were reimbursed in France (in 2006) [9], potentially resulting in less frequent diagnosis (and treatment) of OP among patients in the database prior to 2006. Additionally, 37 % of physicians who participated in the INSTANT study were rheumatologists who may manage patients more proactively than the general practitioners represented in this study. Almost all (97 %) OP patients in a study by Blotman et al. [10] received treatment. However, in that study, GPs recruited women with a diagnosis of OP who had been followed for at least 2 years, perhaps increasing the likelihood of receiving treatment. The GLOW study reported 41 to 50 % of European women aged 55+ with self-reported OP were treated [28], but treatment information was also self-reported. Among those initiating treatment in our study, 93 % received BIS, which is slightly higher than in previous studies [29, 30]. The lower rate of treatment penetration observed in this study compared with previous research may also reflect the methodology used to identify patients with osteoporosis. Patients were included study if they had a diagnosis code for osteoporosis in the claims database used in this study. However, French recommendations for pharmacotherapy also take into consideration other risk factors, including bone mineral density and FRAX score [9], and these metrics were not available in the claims database. Thus, while patients in this study had a diagnosis of osteoporosis recorded in the database, it is possible that they did not meet the criteria for guideline-recommended pharmacotherapy. As such, these results may overestimate the proportion of women who would be considered candidates for treatment according to guideline.

Among all women in this study, mean age was 70.3 years and 11.4 % had evidence of a post-diagnosis GI event. GI conditions are more prevalent in the general elderly population [31] and previous studies of women with low bone density have reported a higher prevalence of GI conditions. For example, in a large scale study of women in Europe (mean age 68.2 years) which included French women, 22.2 % of women had upper GI conditions (gastro-esophageal reflux disease, reflux, dyspepsia) and 11.7 % had lower GI conditions (irritable bowel syndrome, Crohn’s disorder) according to data recorded by site investigators [32]. Our study captured a broader range of upper GI and lower GI conditions but our estimates of GI events were based upon the presence of claims documenting a medical consultation or treatment for GI symptoms. Patients in our study may have had milder GI symptoms for which they did not seek medical attention but chose to self-treat with non-prescription medications. As a result, the proportion of GI events observed in this study and the use of gastro protective agents (limited to prescription drugs only) are likely underestimated.

Although our study sample represented older women, the association between post-diagnosis GI events and reduced odds of treatment initiation that we observed complement previous findings in younger patients. In a study of Israeli women (mean age 65 years), which used methodology comparable to ours, Yu and colleagues observed that a post-diagnosis GI event reduced the odds of treatment initiation by 85 % [33]. Among US women (mean age 66 years), post-diagnosis GI events were associated with 75 % lower likelihood of treatment initiation [34]. Block et al. found that pre-existing GI disease in elderly US women experiencing a fracture was associated with a non-significant trend for lower likelihood of receiving treatment [35]. In our study, we found only moderate differences by pre-index GI events, but found that post-index GI events (i.e., those occurring after OP diagnosis) were significantly associated with non-treatment Other associations included age, disease burden, and pre-index medication use suggesting that our findings are relevant.

Among women who initiated treatment, a post-diagnosis GI event also significantly reduced the likelihood of initiating therapy with BIS vs non-BIS. Raloxifene was the most common non-BIS treatment initiated. Foster et al. also observed that certain pre-treatment GI conditions increased the likelihood of treatment with raloxifene compared with oral BIS in US Medicare and commercial patients, but not in a Medicaid cohort [36]. In our analysis limited to patients who initiated treatment, osteoporosis pharmacotherapy was grouped into two categories: all BIS agents (including oral and IV formulations) and non-BIS agents. As a result of this categorization, agents with distinct pharmacological profiles (e.g., raloxifene vs. calcitonin) and method of administration (e.g., oral BIS vs IV BIS) were grouped together. The models examining the association between GI events and type of treatment initiated (BIS vs non-BIS) lacked adjustment variables for BIS drug formulation (oral vs IV) because an indicator of dosage form was not available in the database extract. Further, the sample size of patients who initiated each non-BIS was too small to make robust estimates of the association between GI events and individual non-BIS treatments. Thus, our findings for the lower likelihood of treatment initiation with BIS vs non-BIS should be interpreted with caution.

There may be several potential reasons for non-treatment of OP. Physicians may not prescribe OP medication because the patient’s risk of fracture is below the threshold for guideline-recommend pharmacotherapy or have other risk factors such as medical conditions or contraindications to therapy that outweigh the benefit of treatment. The patient may also be unwilling to take or comply with physician treatment recommendations because they lack an understanding of OP and risk of fracture, are skeptical of the effectiveness of therapy [14, 15] or have concerns over side effects [16]. GI intolerance has also been observed to affect prescribing behavior [22], especially among those with pre-treatment GI events who are more likely to experience post-treatment GI events [26]. Patients on OP therapy demonstrate worse adherence [37], and are more likely to discontinue [38–40] in the presence of GI events. Our results coupled with previous studies suggest that GI events are also one of the barriers to effective treatment of OP and highlight the need for clinicians to proactively monitor GI events before and after treatment initiation and to manage patients accordingly.

### Strengths and limitations

The strength of this analysis comes from the large, representative database these patients were drawn from. However, there are several limitations that should be considered. Patients with OP were identified from diagnoses on claims. Claims may be missing or miscoded, and claims may represent a screening for a disease, rather than a diagnosis. Clinical results (e.g., T-scores) were not available in the database to confirm the diagnosis of osteoporosis (in France, patients who initiate treatment for osteoporosis do not always undergo a DXA scan in clinical practice). Further, recommendations for initiation of pharmacotherapy are based on patient risk factors. The database used in this analysis lacked bone mineral density test results and FRAX scores. The study relied on a diagnosis of osteoporosis as an indication for pharmacotherapy and, thus, may have overestimated the number and proportion of patients for whom pharmacotherapy should be considered. Claims data cannot distinguish between clinician failure to prescribe treatment and patient failure to fill a prescription. Over-the-counter medications used by patients with osteoporosis (e.g., calcium, Vitamin D) and GI disease (e.g., antacids, proton pump inhibitors) and those provided to the patient by the physician as samples are excluded from the database. The use of claims data limits the identification of GI events to those that resulted in utilization of medical services, which may lead to an under-representation of these events. Further, our analysis considered all GI events as a single class. Future research should examine the potential association between specific types of GI events, (particularly common conditions such as gastro-esophageal reflux disease) and treatment initiation patterns. Certain patient characteristics and attributes of the study population may also limit the generalizability of our data. Variations in treatment rates of osteoporosis between countries have been documented [28]; for this reason, these results may not be applicable to other geographic settings. The results are limited to women aged ≥55 years and those receiving care from primary care practitioners. Patient characteristics such as race and ethnicity that may have an impact on medication treatment are not captured in this study. Despite these limitations, these data reflect “real world” practice of OP treatment from GPs, who are the most frequent prescribers of OP treatment.

## Conclusions

Among women patients aged 55 years or older with a diagnosis of OP, the occurrence of GI events after the OP diagnosis was associated with a lower likelihood of treatment initiation.

## Ethics

No identifiable protected health information was extracted or accessed during the course of the study. Pursuant to the US Health Insurance Portability and Accountability Act. the use of de-identified data does not require Institutional Review Board approval or waiver of authorization [41].

## Additional file

Additional file 1: Codes for identification of fractures and gastro-intestinal events. (DOCX 33 kb)

## Abbreviations

BIS: bisphosphonate
CCI: Charlson Comorbidity Index
CI: confidence interval
EU: European Union
FRAX: World Health Organization Fracture Risk Assessment Tool
GI: gastrointestinal
ICD-10: International Classification of Diseases, Tenth Revision
IV: intravenous
NSAID: non-steroidal anti-inflammatory drug
OP: osteoporosis
OR: odds ratio
SD: standard deviation
US: United States
