# Supplementary material for: Association between gastrointestinal events and osteoporosis treatment initiation in women diagnosed with osteoporosis in France: a retrospective analysis
Source: BMC Musculoskelet Disord. 2016 Apr 30;17:195. doi: 10.1186/s12891-016-1041-8 (PMC4852421; doi:10.1186/s12891-016-1041-8)
Supplement: Additional file 1: — Codes for identification of fractures and gastro-intestinal events. (DOCX 33 kb) [file 12891_2016_1041_MOESM1_ESM.docx]

Codes for identification of fractures and gastro-intestinal events

| **Fracture** | **ICD-10 code** |
| --- | --- |
| Vertebral fracture | S120, S121, S122, S127, S129, S220, S221, S320, S322, T08 |
| Hip fracture | S72 |
| Non-vertebral fracture | S422, S423, S424 |
| Wrist fracture | S52 |

Codes for identification of gastro-intestinal events

| **Gastro-intestinal event** | **ICD-10 code** |
| --- | --- |
| K259 | Gastric ulcer, unspecified as acute or chronic, without hemorrhage or perforation |
| K261 | Acute duodenal ulcer with perforation |
| K260 | Acute duodenal ulcer with hemorrhage |
| K261 | Acute duodenal ulcer with perforation |
| K262 | Acute duodenal ulcer with both hemorrhage and perforation |
| K263 | Acute duodenal ulcer without hemorrhage or perforation |
| K264 | Chronic or unspecified duodenal ulcer with hemorrhage |
| K265 | Chronic or unspecified duodenal ulcer with perforation |
| K266 | Chronic or unspecified duodenal ulcer with both hemorrhage and perforation |
| K267 | Chronic duodenal ulcer without hemorrhage or perforation |
| K269 | Duodenal ulcer, unspecified as acute or chronic, without hemorrhage or perforation |
| K270 | Acute peptic ulcer, site unspecified, with hemorrhage |
| K271 | Acute peptic ulcer, site unspecified, with perforation |
| K272 | Acute peptic ulcer, site unspecified, with both hemorrhage and perforation |
| K273 | Acute peptic ulcer, site unspecified, without hemorrhage or perforation |
| K274 | Chronic or unspecified peptic ulcer, site unspecified, with hemorrhage |
| K275 | Chronic or unspecified peptic ulcer, site unspecified, with perforation |
| K276 | Chronic or unspecified peptic ulcer, site unspecified, with both hemorrhage and perforation |
| K277 | Chronic peptic ulcer, site unspecified, without hemorrhage or perforation |
| K279 | Peptic ulcer, site unspecified, unspecified as acute or chronic, without hemorrhage or perforation |
| K280 | Acute gastrojejunal ulcer with hemorrhage |
| K281 | Acute gastrojejunal ulcer with perforation |
| K282 | Acute gastrojejunal ulcer with both hemorrhage and perforation |
| K283 | Acute gastrojejunal ulcer without hemorrhage or perforation |
| K290 | Acute gastritis |
| K2941 | Chronic atrophic gastritis with bleeding |
| K2951 | Unspecified chronic gastritis with bleeding |
| K296 | Other gastritis |
| K299 | Gastroduodenitis |
| K298 | Duodenitis |
| R1110 | Vomiting, unspecified |
| K30 | Functional dyspepsia |
| K3189 | Other diseases of stomach and duodenum |
| K319 | Disease of stomach and duodenum, unspecified |
| K313 | Pylorospasm, not elsewhere classified |
| K3181 | Angiodysplasia of stomach and duodenum |
| K3182 | Dieulafoy lesion (hemorrhagic) of stomach and duodenum |
| K3189 | Other diseases of stomach and duodenum |
| K319 | Disease of stomach and duodenum, unspecified |
| K631 | Perforation of intestine (nontraumatic) |
| K920 | Hematemesis |
| K921 | Melena |
| K922 | Gastrointestinal hemorrhage, unspecified |
| R112 | Nausea with vomiting, unspecified |
| R110 | Nausea |
| R111 | Vomiting |
| R12 | Heartburn |
| R130 | Aphagia |
| R1310 | Dysphagia, unspecified |
| R1311 | Dysphagia, oral phase |
| R1312 | Dysphagia, oropharyngeal phase |
| R1313 | Dysphagia, pharyngeal phase |
| R1314 | Dysphagia, pharyngoesophageal phase |
| R1319 | Other dysphagia |
| R109 | Unspecified abdominal pain |
| R101 | Abdominal pain |
| R195 | Other fecal abnormalities |
| R933 | Abnormal findings on diagnostic imaging of other parts of digestive tract |
